# Physical exercise and lower psychological distress among rural left-behind adolescents in China: evidence from a moderated serial mediation model

**DOI:** 10.3389/fpsyg.2026.1861638

**Published:** 2026-10-23

**Authors:** Tian Yin, Yanfeng Peng, Xu Liu, Youpeng Zhu, Jiale Wang, Tiande Pan, Changcheng Xie

**Affiliations:** 1 Hezhou University, Hezhou, Guangxi, China; 2 School of Physical Education, Yunnan Minzu University, Kunming, Yunnan, China; 3 School of Physical Education, Xinjiang Normal University, Urumqi, Xinjiang, China

**Keywords:** emotional intelligence, gender moderation, general self-efficacy, physical exercise, psychological distress, rural left-behind adolescents, serial mediation

## Abstract

**Objective:**

This study investigated the association between physical exercise and psychological distress among rural left-behind adolescent students in China. It also assessed the serial mediating effects of general self-efficacy and emotional intelligence and the moderating role of gender in this pathway.

**Methods:**

A total of 1,399 rural left-behind adolescent students in China took part in the survey. They completed the Physical Activity Rating Scale, the General Self-Efficacy Scale, an Emotional Intelligence Scale, and a Psychological Distress Scale. Data were analyzed using descriptive statistics, correlation analysis, serial mediation analysis, and moderated mediation analysis in SPSS 26.0 and AMOS 27.0.

**Results:**

Physical exercise was significantly and negatively correlated with psychological distress. The total effect of physical exercise on psychological distress was significant. After general self-efficacy and emotional intelligence were included in the model, the direct effect of physical exercise on psychological distress remained significant, although reduced in magnitude. The separate indirect effects through general self-efficacy and emotional intelligence were −0.171 and −0.030, respectively, and the serial indirect effect through general self-efficacy and emotional intelligence was −0.063. The total indirect effect was −0.264, accounting for 81.73% of the total effect. In addition, gender significantly moderated the relationship between emotional intelligence and psychological distress, with emotional-intelligence-related indirect pathways being stronger among female students.

**Conclusion:**

Physical exercise was significantly associated with lower psychological distress among rural left-behind adolescent students in China. General self-efficacy and emotional intelligence played significant separate and serial mediating roles in this association, with general self-efficacy showing the larger indirect association. Gender further strengthened the protective pathway centered on emotional intelligence. These findings provide preliminary empirical clues and a theoretical framework for future physical-exercise-based mental health promotion programmes for left-behind adolescents. Longitudinal and experimental studies are needed to verify the temporal and causal directions among physical exercise, general self-efficacy, emotional intelligence, and psychological distress.

## 1 Introduction

Adolescence is a highly sensitive developmental stage during which physical maturation, cognitive restructuring, and emotion-regulation systems undergo rapid changes. Mental health problems are therefore particularly salient across different adolescent populations (Rapee et al., 2019). Psychological distress (PD), which captures non-specific negative emotional states such as nervousness, hopelessness, restlessness, depressed mood, and feelings of worthlessness, is commonly used to assess subclinical but meaningful mental health risks among adolescents (Zhu et al., 2022). Adolescence is also a critical period for the escalation of internalizing problems, during which psychological difficulties tend to show higher prevalence and faster accumulation (Black et al., 2022). From a global public health perspective, adolescent mental health problems constitute a substantial burden. The World Health Organization has reported that approximately one in seven adolescents experiences a mental health problem, with around 14% of individuals aged 10–19 years affected. In this context, PD is useful for identifying adolescents who may be at risk of poor psychological adjustment (World Health Organization, 2024). A meta-analysis further reported that individuals with high PD had a 29% higher risk of mortality than those with low PD (Barry et al., 2020). Thus, PD should not be seen only as temporary emotional discomfort. It may also indicate a longer-term risk to healthy development. Among adolescent students, prolonged PD may affect school engagement, peer relationships, and social functioning, and may increase the likelihood of more serious mental health problems (Counts et al., 2025; Ban et al., 2017). This issue may be especially relevant to left-behind adolescent students who experience parent–child separation during adolescence (Liu et al., 2023). In China, this group is mainly found in rural areas and is often formed when parents migrate for work to improve family income. Many left-behind students therefore grow up with limited parental company, reduced emotional support, and unstable family resources (Qin and Albin, 2010). Prior studies suggest that long-term parent–child separation, insufficient parental support, weak peer support, and negative peer interactions may create an unfavorable social–emotional environment. Such conditions may weaken emotion regulation and stress recovery, thereby increasing the risk of PD, depressive symptoms, and self-harm behaviors (Wen and Lin, 2012). Therefore, identifying protective factors against PD among rural left-behind adolescent students is important for both theory and practice, especially for school-based mental health promotion and early intervention.

### 1.1 Physical exercise and psychological distress

Physical exercise (PE) refers to planned, structured, and repeated physical activity aimed at maintaining or improving physical fitness. It is a changeable behavior and can be promoted through school-based and community-based programs, making it a useful target for adolescent mental health intervention (Lukács et al., 2020; Peng B. et al., 2025; Peng J. et al., 2025). Recent meta-analytic evidence suggests that regular PE is associated with better mental health among adolescents, particularly with fewer depressive, anxiety, and stress-related symptoms (Ruiz-Ranz and Asín-Izquierdo, 2025). From the view of self-determination theory, PE may help meet adolescents’ needs for autonomy, competence, and relatedness. In this way, PE may support positive emotional experiences, strengthen adaptive motivation, and reduce negative affect and psychological discomfort (Owen et al., 2014). Among rural left-behind children, PE has been shown to be positively associated with more favorable psychological and social adjustment indicators, suggesting that it may serve as a meaningful protective resource for this vulnerable population (Li and Ren, 2024). This issue deserves attention because left-behind adolescent students are more likely to experience PD and often lack stable psychosocial support. However, many adolescents do not engage in enough PE. Globally, approximately 81% of adolescents aged 11–17 years fail to meet physical activity recommendations, with insufficient activity reported in about 85% of girls and 78% of boys (Guthold et al., 2020). Similar concerns have been reported in China, where national data show that moderate-to-vigorous physical activity among school-aged children and adolescents remains low, and fewer than one-third meet the recommended level (Fan and Cao, 2017). Recent equity-focused research further indicates that access to physical activity is uneven across social and geographic groups, with rural, migrant, and left-behind children facing greater barriers to regular PE participation (Li et al., 2025). Therefore, examining PE in relation to PD among left-behind adolescent students may help clarify how a modifiable behavior is associated with adolescent mental health. It may also offer evidence for school-based prevention and support in rural settings. Improving PE opportunities in rural schools may support both physical development and psychological adjustment during adolescence (Zhuang and Wu, 2024). Based on this rationale, the following hypothesis was proposed:

*H1*: PE is significantly and negatively associated with PD.

### 1.2 The mediating role of general self-efficacy

General self-efficacy (GSE) refers to individuals’ generalized belief in their capacity to organize and execute actions required to cope with external challenges and internal stressors (Bandura, 1978). Self-efficacy theory suggests that GSE beliefs shape how individuals appraise stressful situations, select coping strategies, and respond emotionally, and are therefore closely related to psychological adjustment (Bandura, 1977). In the context of GSE, PE should not be understood merely as bodily activity, but also as a behavioral process that may repeatedly provide mastery experiences, self-verification, and competence feedback (Hagger, 2019). Through goal attainment, skill mastery, improved physical fitness, and an enhanced sense of bodily control during regular PE, adolescents may gradually develop more positive judgments of their own competence, which constitute an important psychological basis for GSE. Peng B. et al. (2025) and Peng J. et al. (2025) found a significant positive association between PE and adolescents’ GSE in a sample of 1,613 adolescents from 15 Chinese provinces (Peng B. et al., 2025; Peng J. et al., 2025). Meanwhile, GSE is closely related to psychological functioning. This interpretation is consistent with Bandura’s social cognitive theory: individuals with higher levels of GSE may be more likely to view stressful events as manageable and controllable challenges, and thus experience fewer negative emotional responses such as anxiety and helplessness. In contrast, individuals with lower GSE may be more likely to experience a sense of incompetence when facing stress and setbacks, accompanied by PD-related responses such as hopelessness, restlessness, depressed mood, and feelings of worthlessness (Zhou and Guo, 2006; Xing et al., 2023). Previous studies have also found that GSE is positively associated with adolescent well-being and positive adjustment, and negatively associated with negative emotional outcomes such as anxiety and depression (Andretta and McKay, 2020). For left-behind adolescent students who experience prolonged parent–child separation, limited emotional support, and greater exposure to adverse life events, this competence-based coping resource may be particularly important. Related evidence also suggests that, among left-behind children or other high-risk adolescent populations, higher GSE is associated with better mental health outcomes (Liao et al., 2023). Thus, GSE is more closely aligned with a cognitive belief in one’s competence than with emotion-processing ability in specific situations. This distinction also provides the basis for differentiating GSE from EI in the following section: the former mainly reflects generalized cognitive competence judgments, whereas the latter emphasizes the perception, understanding, and regulation of emotional information. Based on this rationale, the following hypothesis was proposed:

*H2*: GSE mediates the relationship between PE and PD.

### 1.3 The mediating role of emotional intelligence

When facing academic pressure, interpersonal conflict, and family adversity, adolescents’ levels of PD may be related not only to whether they believe they can cope with stress, but also to how accurately they identify, understand, express, and regulate their emotions. Emotional intelligence (EI) is generally viewed as a psychological ability related to emotional information processing and emotion regulation, reflecting individuals’ capacity to perceive, understand, use, and manage emotions (Salovey et al., 2004). Unlike GSE, which represents a generalized cognitive competence belief, EI emphasizes the identification, interpretation, and regulation of emotional cues in specific emotional contexts. It can therefore be regarded as another relatively distinct emotional psychological resource pathway linking PE and PD. Previous research suggests that higher EI is often associated with better psychological adjustment in adolescents, indicating that EI may serve as an important emotional psychological resource (Davis and Humphrey, 2012). In stressful contexts, individuals with higher EI may be more able to recognize emotional cues in themselves and others, and to understand and regulate negative emotions in more adaptive ways, thereby reducing emotional dysregulation and the risk of PD (Luebbers et al., 2007; Li et al., 2024). Conversely, adolescents with lower EI may be more prone to negative rumination, impulsive reactions, and difficulties in emotion regulation when facing academic pressure, interpersonal conflict, or family adversity, which may further manifest as anxiety, depression, and broader psychological maladjustment (Resurrección et al., 2014). A cross-sectional study of 467 students also found a significant negative association between EI and PD (Zhang et al., 2016). For left-behind adolescent students who experience prolonged parent–child separation, limited emotional support, and greater exposure to negative peer interactions, emotion processing and regulation may be especially important for adjustment (Lin and Guo, 2024). From the perspective of self-determination theory, when an activity continually satisfies individuals’ needs for relatedness, competence, and autonomy, it is more likely to be associated with positive emotional experiences and psychological adjustment (Owen et al., 2014). Thus, PE may not only be related to physical functioning but also provide social and emotional experiences involving peer interaction, rule following, competition and cooperation, and emotional expression. In exercise contexts, adolescents need to identify their own emotions, understand feedback from others, and regulate emotional responses during competition, cooperation, or frustration. These experiences may be associated with EI levels, a view also supported by evidence linking PE to adolescents’ EI (Yang et al., 2024). Accordingly, EI may be understood as an emotional mediation pathway linking PE and PD. Based on this rationale, the following hypothesis was proposed:

*H3*: EI mediates the relationship between PE and PD.

### 1.4 The serial mediating roles of general self-efficacy and emotional intelligence

Beyond their independent mediating roles, GSE and EI may also be linked in a theoretically meaningful serial pathway. Prior research has shown a significant positive association between GSE and EI among secondary school students, suggesting that these two psychological resources are not entirely independent but may jointly contribute to adolescent psychological adjustment (Salavera et al., 2017). Based on self-efficacy theory and broaden-and-build theory, the present study positioned GSE before EI. Specifically, GSE represents a generalized cognitive competence belief, reflecting individuals’ overall judgment of whether they can cope with difficulties and manage stressful situations. EI, by contrast, reflects emotional information processing and regulation abilities, including the ability to identify, understand, use, and manage emotions. Self-efficacy theory suggests that individuals’ judgments of their coping capacity may shape their stress appraisal, effort investment, coping choices, and emotion regulation tendencies (Bandura, 1977). Broaden-and-build theory further proposes that positive emotions and positive psychological resources can broaden individuals’ cognitive and behavioral repertoires and, over time, build more enduring personal resources (Fredrickson, 2001). Positive affect and self-determination processes may also jointly foster positive psychological resources such as GSE and EI (Stanley and Schutte, 2023). Accordingly, higher GSE may make adolescents more willing to notice, interpret, and regulate their emotions when facing stress or negative affect, thereby providing a cognitive basis for the activation or development of EI. For left-behind adolescent students, this pathway has clear contextual relevance. Prolonged parent–child separation, limited emotional support, and greater exposure to adverse life events may make these adolescents more dependent on internal psychological resources when coping with academic pressure, interpersonal conflict, and negative emotions (Zhuang et al., 2025). Related research also suggests that left-behind students often rely on emotional self-regulation, peer support, and other internal or proximal resources to cope with negative emotions and academic difficulties (Lin et al., 2025). In this context, the role of PE may not be limited to directly buffering negative emotions; rather, PE may function as an external behavioral resource associated with the accumulation and transformation of internal psychological resources. Che et al. (2026) suggested that PE may provide a practical basis for the gradual accumulation of internal psychological resources. Studies of secondary school students have also found that PE is positively associated with GSE and stress self-management, and that moderate-to-vigorous PE is associated with EI and may be linked to lower perceived stress through the mediating role of EI (Li et al., 2024). Other studies further indicate that GSE, EI, and student stress or stressful-task performance are closely connected, suggesting that cognitive competence beliefs and emotional processing abilities may jointly contribute to stress adaptation (Navarro-Mateu et al., 2020; Udayar et al., 2020). Thus, the GSE → EI pathway can be understood as a theoretically grounded link between cognitive competence beliefs and emotional processing ability. However, this pathway does not rule out the possibility that EI may precede GSE or that the two may influence each other reciprocally. Although the present study positioned GSE before EI based on self-efficacy theory and broaden-and-build theory, this ordering should not be interpreted as definitive evidence of temporal sequence or causal direction. Based on this rationale, the following hypothesis was proposed:

*H4*: GSE and EI serially mediate the relationship between PE and PD.

### 1.5 The moderating role of gender in the relationship between emotional intelligence and psychological distress

Previous research has shown that both EI and PD vary by gender. Developmental research on EI has found that girls generally score higher than boys on overall EI and several of its branches (Megías-Robles et al., 2024). Meanwhile, a cross-sectional study of 566,829 adolescents across 73 countries showed that girls, on average, reported higher levels of internalizing mental health problems, with this gender difference being particularly evident for PD (Campbell et al., 2021). However, the focus of the present study is not whether gender affects the mean levels of EI or PD, but whether the association between EI and PD differs by gender. In other words, gender may primarily moderate the EI → PD emotion-related resource pathway, rather than simply altering the overall levels of PE, EI, or PD. From the perspective of emotion regulation strategies, girls are generally more likely to use emotion-focused coping and to engage in rumination when facing stress. A meta-analysis of gender differences in coping showed that women more frequently use coping strategies involving the expression of one’s own or others’ emotions, including seeking emotional support, ruminating about problems, and positive self-talk (Tamres et al., 2002). Nolen-Hoeksema (2012) also noted that women’s greater tendency toward rumination partly explains their higher risk of depression and anxiety. Thus, when girls experience higher emotional arousal and more frequent emotion-processing demands, EI may become a particularly important protective resource. Higher EI may help individuals identify, understand, and regulate negative emotions more accurately, thereby weakening the links of rumination and emotional dysregulation with PD. For left-behind adolescent students, this gender difference may have particular contextual relevance. Prolonged parent–child separation, limited emotional support, and negative peer interactions may make left-behind students more dependent on proximal emotion-regulation resources to maintain psychological adjustment (Lin et al., 2025). In this context, girls’ greater emotional attention and emotion-processing tendency may make them more vulnerable to the accumulation of negative emotions; for the same reason, however, the negative association between EI and PD may be stronger among girls. Thus, gender differences may not primarily lie in the direct association between PE and PD, but rather in the strength of the protective association between EI and PD. Based on this rationale, the following hypothesis was proposed:

*H5*: Gender moderates the relationship between EI and PD.

### 1.6 The present study

Previous research has helped explain the link between PE and adolescent mental health, but several questions remain. First, most studies have examined depression, anxiety, stress, or general mental health, whereas PD has received less attention as a broader and continuous marker of psychological risk. Second, although prior work has separately reported associations between PE and GSE, PE and EI, and GSE and EI, few studies have tested these variables within the same explanatory model. Third, evidence has mainly come from general adolescent samples, leaving rural left-behind adolescent students less studied. Finally, although EI and PD both show gender differences, it remains unclear whether gender changes the EI–PD association and whether this effect extends to the indirect pathway linking PE to PD. To address these gaps, the present study focused on rural left-behind adolescent students in China and examined PD as the key outcome. Specifically, we tested whether GSE and EI served as independent and serial mediators between PE and PD, and whether gender moderated the EI–PD pathway and the corresponding indirect effects. This study therefore aimed to provide a more nuanced understanding of the psychological resource pathway linking PE to PD and to offer evidence for mental health promotion among Chinese left-behind adolescents (see Figure 1).

**Figure 1:**
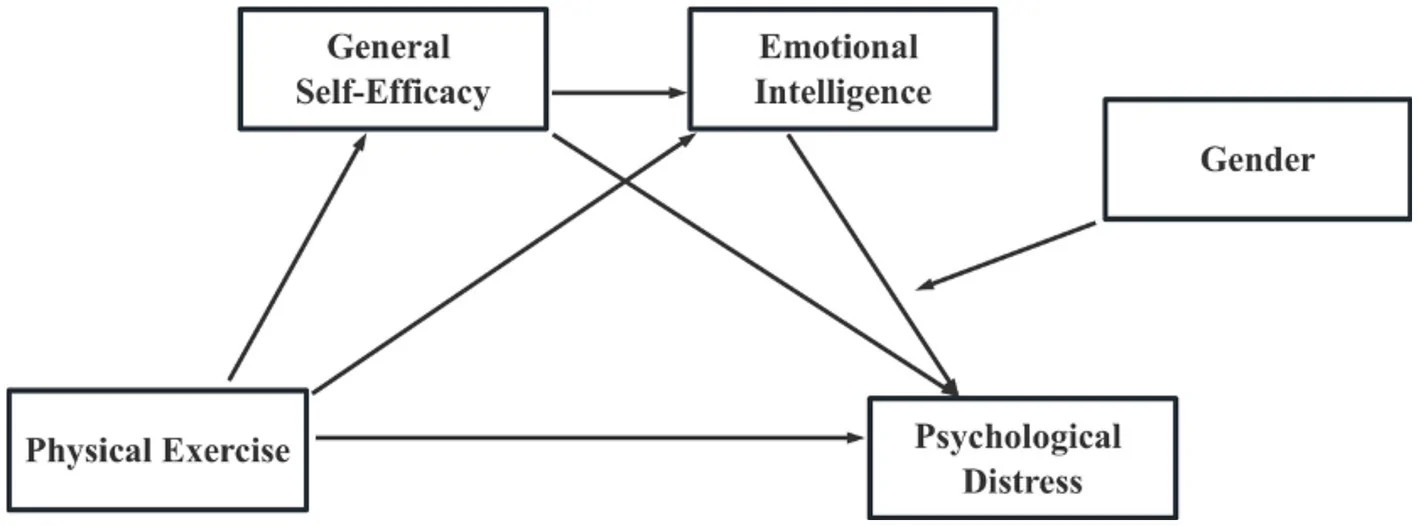
Hypothesized moderated serial mediation model.

Based on the above rationale, the following hypotheses were proposed:

*H1*: PE is significantly and negatively associated with PD.

*H2*: GSE mediates the relationship between PE and PD.

*H3*: EI mediates the relationship between PE and PD.

*H4*: GSE and EI serially mediate the relationship between PE and PD.

*H5*: Gender moderates the relationship between EI and PD.

## 2 Methods

### 2.1 Participants

Using convenience sampling, we conducted a questionnaire survey from February 28 to March 15, 2025, in 23 rural secondary schools located in Dali City, Yunnan Province; Qianxinan Buyi and Miao Autonomous Prefecture, Guizhou Province; and Liuzhou City, Guangxi Zhuang Autonomous Region, China. Because all participating schools were located in rural areas of western China, the sample has a clear regional specificity, and the generalizability of the findings should be interpreted with caution. A total of 3,656 questionnaires were distributed on site to screen students’ left-behind status and collect basic demographic information. Students who were not identified as left-behind adolescent students were not included in the subsequent statistical analyses. Among the screened students, 1,614 met the eligibility criteria for left-behind adolescent students and completed the core questionnaire.

Before data collection, the study purpose and survey content were explained to participants’ guardians through the head teachers. Participation was voluntary, and written informed consent was obtained from both the students and their guardians. During the survey, trained researchers read standardized instructions to all participants, explaining the study purpose, the principles of data confidentiality, and the intended use of the data. Participants were also informed that participation was entirely voluntary, that they could discontinue the survey at any time, and that the survey involved no known risk. The questionnaires were administered in classroom groups using paper-and-pencil forms, which were distributed and collected on site.

This study was reviewed and approved before its initiation by the Science and Technology Ethics Committee of Yunnan Minzu University (approval number: YXLL2025001). The inclusion criteria were: (1) being a left-behind adolescent student; (2) being formally enrolled in Grades 7–12 at one of the participating schools; and (3) having sufficient reading and writing ability to complete the questionnaire independently. The exclusion criteria were: (1) not meeting the definition of a left-behind adolescent student; (2) showing obvious patterned responses or leaving large sections of the questionnaire blank; and (3) having 10% or more missing data on key variables. After data screening, 1,399 valid questionnaires were retained, representing 86.68% of the eligible left-behind student sample. The final sample consisted of 736 boys (52.61%) and 663 girls (47.39%), with a mean age of 14.58 years (*SD* = 1.79).

### 2.2 Measures

#### 2.2.1 Physical exercise

PE was measured using the Physical Activity Rating Scale-3 (PARS-3), which was revised for use in Chinese populations (Liang, 1994). The scale comprises three items assessing exercise intensity, duration, and frequency, with each item rated on a 5-point scale. Following the standard scoring method, the total PE score was calculated as exercise intensity × (exercise duration − 1) × exercise frequency, yielding a possible score ranging from 0 to 100. Higher scores indicate greater participation in physical exercise. Because the three items represent distinct components of exercise behavior and are combined using a multiplicative scoring formula, Cronbach’s *α* was not calculated for this scale.

#### 2.2.2 Psychological distress

PD was assessed using the 9-item version of the Kessler Psychological Distress Scale (K-9), which has shown good psychometric properties in adolescent samples (Larzabal-Fernandez et al., 2024). The scale assesses non-specific symptoms of PD experienced during the past month, including nervousness, hopelessness, restlessness, depressed mood, and feelings of worthlessness. It consists of nine items rated on a 4-point Likert scale ranging from 1 = never to 4 = always. Total scores range from 9 to 36, with higher scores indicating higher levels of PD. In the present study, Cronbach’s *α* for this scale was 0.892. The 9-item Kessler scale has been reported to show good factorial validity, gender invariance, and adequate internal consistency in adolescents.

#### 2.2.3 General self-efficacy

GSE was measured using the General Self-Efficacy Scale, which assesses individuals’ generalized beliefs about their ability to cope with difficulties and complete demanding tasks (Schwarzer and Jerusalem, 1995). It consists of 10 items. In the version administered in the present study, the items were rated on a 5-point Likert scale ranging from 1 = completely incorrect to 5 = completely correct. Total scores range from 10 to 50, with higher scores reflecting higher levels of GSE. In the present study, Cronbach’s α for this scale was 0.887.

#### 2.2.4 Emotional intelligence

EI was assessed using the Self-Rated Emotional Intelligence Scale–Revised, a 19-item self-report measure based on Mayer and Salovey’s emotional intelligence framework (Brackett et al., 2006). The scale is based on Mayer and Salovey’s ability model of emotional intelligence and assesses individuals’ perceived ability to perceive, use, understand, and manage emotions. It contains 19 items rated on a 5-point Likert scale ranging from 1 = very inaccurate to 5 = very accurate. Total scores range from 19 to 95, with higher scores indicating higher levels of EI. In the present study, Cronbach’s α for this scale was 0.897. Previous descriptions of the instrument identify it as a 19-item self-report scale mapping onto emotional abilities assessed by the MSCEIT, with responses made on a 5-point scale.

### 2.3 Covariates

Previous research suggests that gender, grade level, and academic performance may be associated with adolescents’ PE, GSE, EI, and PD, and therefore may confound estimates of the relationships among the main study variables. Gender differences have been observed in adolescent physical activity, self-efficacy-related pathways, and mental health outcomes, while academic performance is also closely linked to adolescents’ psychological well-being and distress (Monzonís-Carda et al., 2025). Therefore, grade level and academic performance were included as covariates in the statistical analyses to reduce potential confounding and to provide a more accurate test of the association between PE and PD, as well as the serial mediating roles of GSE and EI.

### 2.4 Data processing and statistical analysis

All statistical analyses were conducted using SPSS 26.0 and AMOS 27.0. Descriptive statistics were calculated based on the raw scores of each variable. Before the mediation and moderated mediation analyses, all continuous variables entered into the regression models were standardized. Given that the PARS-3 total score was calculated using a multiplicative scoring method, we further examined the distributional characteristics of the PE total score before conducting the regression analyses. The normality tests showed that the PE total score significantly deviated from a normal distribution, as indicated by the Shapiro–Wilk test (W = 0.950, *p* < 0.001) and the Kolmogorov–Smirnov test (D = 0.115, *p* < 0.001). However, descriptive distributional indices indicated that the PE total score showed only mild positive skewness, with a skewness value of 0.450 and a kurtosis value of −0.726. Thus, the distribution did not show severe positive skewness or an extreme long-tailed pattern of high scores. Further inspection of the log-transformed score showed that the distribution instead had stronger negative skewness, with a skewness value of −1.502, and the Shapiro–Wilk test remained significant (W = 0.852, *p* < 0.001). Therefore, to preserve the interpretability of the standard PARS-3 scoring method and comparability with previous studies, the original PE total score was retained in the main analyses, and no log transformation was applied. To examine the structural validity of the measurement model, confirmatory factor analysis was conducted using AMOS 27.0. Given the relatively large number of items in the GSE, EI, and PD scales, and because the present study focused on construct-level structural relationships rather than item-level subdimensions, item parceling was used to construct latent variable indicators before confirmatory factor analysis (Little et al., 2002; Matsunaga, 2008). PE was treated as an observed composite score calculated according to the standard PARS-3 scoring formula and was therefore not modeled as a reflective latent construct in the measurement model. Pearson correlation analysis was then conducted to examine associations among the key variables. Finally, grade level and academic performance were included as covariates, PROCESS Model 6 and Model 87 were used to test the serial mediation effect and moderated mediation effect, respectively, and 5,000 bootstrap resamples were used to estimate indirect effects and their 95% confidence intervals, thereby reducing reliance on the normality assumption for indirect-effect testing.

## 3 Results

### 3.1 Common method bias test

Because PE, GSE, EI, and PD were all assessed using self-report questionnaires completed by the same participants at the same time point, the data may be susceptible to common method bias. As a preliminary diagnostic procedure, we conducted Harman’s single-factor test by entering all items of the core study variables into an exploratory factor analysis (Podsakoff et al., 2003). The unrotated factor solution yielded several factors with eigenvalues greater than 1, and the first factor accounted for 29.66% of the total variance, which did not exceed the commonly used threshold. This result suggests that severe common method bias was not evident in the present data. Nevertheless, Harman’s single-factor test is only a coarse preliminary diagnostic approach and cannot fully rule out the possibility of common method bias arising from single-source, self-report measurement. Therefore, the findings should still be interpreted cautiously in light of the study design and its limitations.

### 3.2 Descriptive statistics and correlation analysis

Table 1 presents the means, standard deviations, and Pearson correlation coefficients for the main study variables. The mean PE score was 32.08, the mean GSE score was 35.50, the mean EI score was 60.77, and the mean PD score was 17.59. These descriptive results are broadly consistent with previous research suggesting substantial individual differences in exercise participation, psychological resources, and mental health adjustment among rural left-behind adolescents (Li and Ren, 2024; Lin et al., 2025; Zhuang et al., 2025). In addition, PE was positively correlated with GSE (*r* = 0.427, *p* < 0.01) and EI (*r* = 0.312, *p* < 0.01), and negatively correlated with PD (*r* = −0.333, *p* < 0.01). GSE was positively correlated with EI (*r* = 0.536, *p* < 0.01). Both GSE (*r* = −0.602, *p* < 0.01) and EI (*r* = −0.547, *p* < 0.01) were negatively correlated with PD. Overall, the directions of these correlations were consistent with the theoretical expectations and provided preliminary support for the subsequent serial mediation and moderated mediation analyses.

**Table 1:** Correlation and descriptive statistics.

Variable	PE	GSE	EI	PD
PE	1			
GSE	0.427**	1		
EI	0.312**	0.536**	1	
PD	−0.333**	−0.602**	−0.547**	1
*M*	32.08	35.50	60.77	17.59
*SD*	21.42	8.63	14.49	5.91

**p < 0.01.

### 3.3 Reliability and validity analysis

To examine the structural and convergent validity of the scales, confirmatory factor analysis was conducted using AMOS 27.0. Given the relatively large number of items in the GSE, EI, and PD scales, and because the present study focused primarily on the overall role of each construct in the structural model rather than item-level subdimensions within each scale, item parceling was used to construct latent variable indicators before model estimation. The parceling procedure followed an item-to-construct balancing strategy: items were ranked according to their corrected item-total correlations with the corresponding scale score and then assigned to different parcels to balance the measurement strength of each parcel as much as possible. Each parcel score was calculated as the mean of the items included in that parcel (Little et al., 2002; Matsunaga, 2008). It should be noted that item parceling may obscure item-level multidimensionality or local misfit. Therefore, this method was used only when the scales had a clear theoretical basis, showed good internal consistency, and the research focus was on construct-level relationships. The PARS-3 was not parceled. The results showed (see Table 2) that the GSE scale had good model fit, the EI scale had acceptable model fit, and the PD scale had good model fit. In addition, both the overall measurement model and the moderated serial mediation structural model reached acceptable fit. According to commonly used fit criteria, CFI/TLI values close to or above 0.95, RMSEA values close to or below 0.06, and SRMR values close to or below 0.08 are generally considered to indicate good or acceptable model fit (Hu and Bentler, 1999). Regarding convergent validity, GSE, EI, and PD showed acceptable composite reliability and convergent validity. CR and AVE were therefore reported for these reflective measurement scales (Fornell and Larcker, 1981). PE was treated as an observed composite score calculated according to the standard PARS-3 scoring formula and was not evaluated using CR or AVE.

**Table 2:** Reliability, validity, and model fit indices.

Scale/model	χ^2^/df	CFI	TLI	RMSEA	SRMR	CR	AVE
GSE	4.142	0.970	0.965	0.038	0.034	0.868	0.570
EI	4.435	0.982	0.977	0.067	0.016	0.846	0.595
PD	1.842	0.986	0.987	0.024	0.019	0.913	0.541
Overall measurement model	4.164	0.958	0.947	0.059	0.056		
Structural model (moderated chain mediation)	4.232	0.973	0.964	0.058	0.059		

### 3.4 Serial mediation analysis

The total effect of PE on PD was significant (*β* = −0.323, 95% CI [−0.372, −0.273], *p* < 0.001). After GSE and EI were entered into the model, the direct effect of PE on PD remained significant but decreased in size (*β* = −0.059, 95% CI [−0.103, −0.015], *p* = 0.009), accounting for 18.27% of the total effect. The total indirect effect was significant (*β* = −0.264, 95% CI [−0.300, −0.229], *p* < 0.001), accounting for 81.73% of the total effect.

Among the specific indirect effects, the pathway through GSE was the strongest (*β* = −0.171, 95% CI [−0.204, −0.140], *p* < 0.001), explaining 52.94% of the total effect. The serial pathway through GSE and EI was also significant (*β* = −0.063, 95% CI [−0.078, −0.050], *p* < 0.001), explaining 19.50%. The pathway through EI was smaller but remained significant (*β* = −0.030, 95% CI [−0.047, −0.015], *p* < 0.001), explaining 9.29%.

These findings indicate that GSE and EI played significant mediating roles in the association between PE and PD. In the baseline serial mediation model, PE was associated with lower PD both directly and indirectly through GSE and EI, with the GSE pathway representing the strongest mediating mechanism (Table 3).

**Table 3:** Serial mediation effects of PE on PD.

Effect	*β*	*SE* / Boot*SE*	LLCI	ULCI	Effect size (%)
Total effect	−0.323***	0.025	−0.372	−0.273	100
Direct effect	−0.059**	0.022	−0.103	−0.015	18.27
Total indirect effect	−0.264***	0.018	−0.300	−0.229	81.73
PE → GSE → PD	−0.171***	0.016	−0.204	−0.140	52.94
PE → EI → PD	−0.030**	0.008	−0.047	−0.015	9.29
PE → GSE → EI → PD	−0.063***	0.007	−0.078	−0.050	19.50

**p < 0.01, ***p < 0.001.

### 3.5 Gender moderation analysis

As shown in Table 4, after controlling for grade level and academic performance, PE was positively associated with GSE (*β* = 0.418, 95% CI [0.370, 0.466], *p* < 0.001) and EI (*β* = 0.099, 95% CI [0.050, 0.147], *p* < 0.001). GSE was also positively associated with EI (*β* = 0.489, 95% CI [0.440, 0.538], *p* < 0.001). In the outcome model, both GSE (*β* = −0.409, 95% CI [−0.458, −0.360], *p* < 0.001) and EI (*β* = −0.171, 95% CI [−0.299, −0.044], *p* = 0.008) were negatively associated with PD, whereas the direct effect of PE on PD was not significant (*β* = −0.031, 95% CI [−0.077, 0.015], *p* = 0.181).

**Table 4:** Moderation effects in the serial mediation model.

Outcome variable	Predictor	*β*	*SE*	*t*	*p*	LLCI	ULCI
GSE	PE	0.418***	0.024	17.191	<0.001	0.370	0.466
	Grade	−0.017	0.019	−0.931	0.352	−0.053	0.019
	Academic	0.102**	0.037	2.761	0.006	0.029	0.174
EI	PE	0.099***	0.025	3.967	<0.001	0.050	0.147
	GSE	0.489***	0.025	19.700	<0.001	0.440	0.538
	Grade	−0.055**	0.017	−3.212	0.001	−0.089	−0.021
	Academic	0.035	0.034	1.014	0.311	−0.032	0.102
PD	PE	−0.031	0.023	−1.337	0.181	−0.077	0.015
	GSE	−0.409***	0.025	−16.341	<0.001	−0.458	−0.360
	EI	−0.171**	0.065	−2.640	0.008	−0.299	−0.044
	Gender	0.159***	0.042	3.754	<0.001	0.076	0.242
	EI × Gender	−0.092*	0.040	−2.278	0.023	−0.170	−0.013
	Grade	−0.028	0.015	−1.821	0.069	−0.058	0.002
	Academic	−0.067*	0.031	−2.193	0.029	−0.127	−0.007
PD ← EI (Boys)	—	−0.263	0.031	−8.402	<0.001	−0.324	−0.202
PD ← EI (Girls)	—	−0.354	0.031	−11.398	<0.001	−0.415	−0.293

*p < 0.05, **p < 0.01, ***p < 0.001.

The interaction between EI and gender was significant (*β* = −0.092, *SE* = 0.040, *t* = −2.278, *p* = 0.023, 95% CI [−0.170, −0.013]), indicating that gender moderated the association between EI and PD. Simple slope analysis showed that EI was negatively associated with PD among both boys (*β* = −0.263, *SE* = 0.031, *p* < 0.001, 95% CI [−0.324, −0.202]) and girls (*β* = −0.354, *SE* = 0.031, *p* < 0.001, 95% CI [−0.415, −0.293]). These findings suggest that gender significantly conditioned the protective association between EI and PD, with EI showing a stronger negative association with PD among girls than among boys (Figure 2).

**Figure 2:**
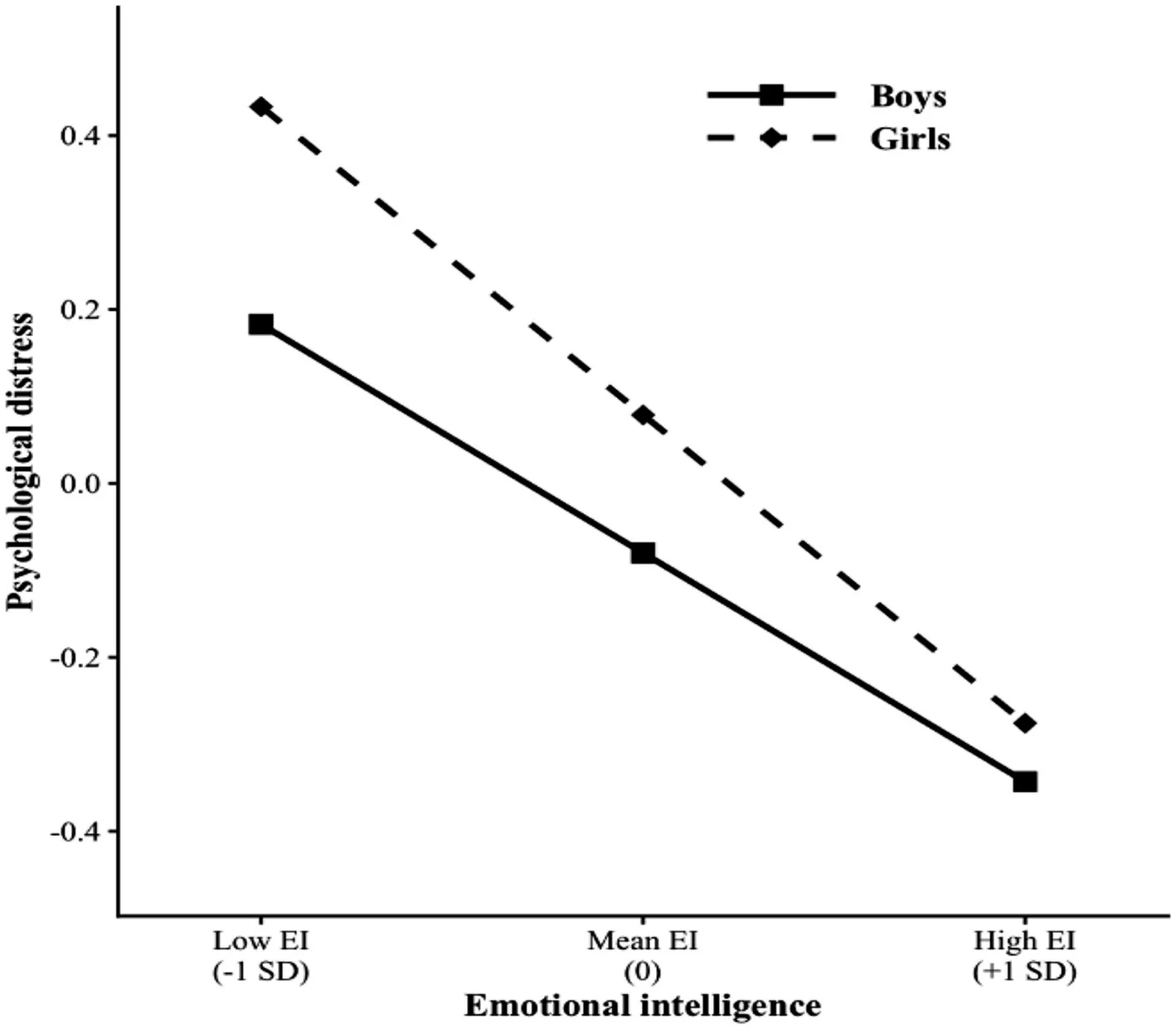
Moderating effect of gender on the association between EI and PD.

### 3.6 Moderated mediation analysis

After controlling for grade level and academic performance, the indirect effect of PE on PD through GSE was significant (*β* = −0.171, 95% bootstrap CI [−0.204, −0.140]). In contrast, the indirect pathways involving EI varied by gender. Specifically, the indirect effect of PE on PD through EI was significant among both boys (*β* = −0.026, 95% CI [−0.042, −0.013]) and girls (*β* = −0.035, 95% CI [−0.055, −0.018]). Similarly, the serial indirect effect through GSE and EI was significant among both boys (*β* = −0.054, 95% CI [−0.070, −0.040]) and girls (*β* = −0.072, 95% CI [−0.091, −0.056]). Both EI-related indirect effects were stronger among girls than among boys. Further analysis of the index of moderated mediation showed that gender significantly moderated the PE → EI → PD pathway (Index = −0.009, 95% CI [−0.018, −0.001]) and the PE → GSE → EI → PD serial pathway (Index = −0.019, 95% CI [−0.034, −0.003]) (see Figure 3). These results indicate that, after accounting for grade level and academic performance, gender primarily moderated the EI-related indirect pathways from PE to PD, with stronger indirect effects observed among girls (Tables 5, 6).

**Figure 3:**
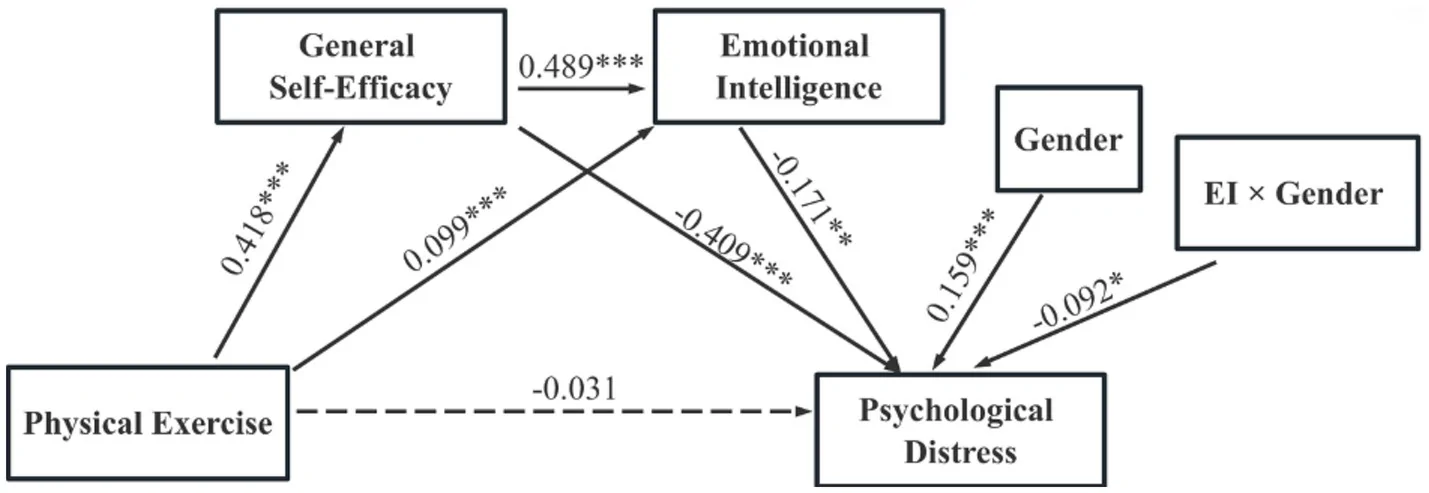
Final moderated serial mediation model with standardized path coefficients. *p < 0.05, **p < 0.01, ***p < 0.001.

**Table 5:** Indirect effects and conditional indirect effects of physical exercise on psychological distress.

Indirect path	Gender	*β*	Boot*SE*	BootLLCI	BootULCI
PE → GSE → PD	Overall	−0.171***	0.016	−0.204	−0.140
PE → EI → PD	Boys	−0.026**	0.008	−0.042	−0.013
	Girls	−0.035***	0.009	−0.055	−0.018
PE → GSE → EI → PD	Boys	−0.054***	0.008	−0.070	−0.040
	Girls	−0.072***	0.009	−0.091	−0.056

**p < 0.01, ***p < 0.001.

**Table 6:** Index of moderated mediation.

Indirect path	Index	BootSE	BootLLCI	BootULCI
PE → EI → PD	−0.009*	0.004	−0.018	−0.001
PE → GSE → EI → PD	−0.019*	0.008	−0.034	−0.003

*p < 0.05.

## 4 Discussion

Building on previous research on the role of PE in adolescent mental health, this study further examined the association between PE and PD among left-behind adolescent students, and tested the mediating roles of GSE and EI as well as the moderating effect of gender on the EI-related pathway (Zhao et al., 2025). The results showed that PE was not only significantly and negatively associated with PD, but also indirectly related to PD through both GSE and EI. In addition, GSE and EI formed a significant serial mediation pathway in the association between PE and PD, while gender further moderated the negative association between EI and PD. These findings are broadly consistent with previous evidence suggesting that PE is associated with fewer negative emotional responses in adolescents and that GSE and emotional competence represent important protective psychological resources (Raimondi et al., 2025; Zhang et al., 2022). More importantly, the significant serial mediation pathway suggests that cognitive belief resources and emotional regulation resources may not be isolated pathways. Instead, they may be involved in a possible cognitive-emotional association pattern related to adolescent adjustment, linking competence appraisal with emotional processing (Jiang, 2024). The moderating role of gender in the EI–PD association further suggests that the protective association of EI is not entirely homogeneous across gender groups, but varies in strength and condition. Overall, these findings contribute to a more nuanced understanding of how PE may be linked to emotional adjustment among left-behind adolescent students through cognitive and emotional psychological resources, and they also provide preliminary theoretical implications for future mental health promotion programmes in this population.

### 4.1 Physical exercise and psychological distress

This study found that PE was significantly and negatively associated with PD among left-behind adolescent students, indicating that higher levels of PE were related to lower levels of PD. This finding is consistent with recent meta-analytic evidence showing that PE may help reduce negative emotional symptoms in adolescents (Yan et al., 2025). For left-behind adolescent students facing long-term parent–child separation, PE may serve not only as physical activity but also as a setting for skill mastery, peer contact, and emotional release. The mediation results provided further detail. In the baseline serial mediation model, the direct effect of PE on PD remained significant but small (*β* = −0.059, *p* = 0.009), whereas the total indirect effect accounted for 81.73% of the total effect. This suggests that the PE–PD association was partly accounted for by psychological pathways involving GSE and EI. After grade level, academic performance, gender, and the EI × Gender interaction were included, the direct effect of PE on PD became nonsignificant (*β* = −0.031, *p* = 0.181). This pattern indicates that the independent association between PE and PD was weakened when cognitive resources, emotional resources, and gender differences were considered together. Therefore, PE may be linked to lower PD mainly through GSE, EI, and EI-related conditional indirect pathways. Its role should not be interpreted as a simple direct buffering association with PD among left-behind adolescent students. Instead, it may reflect a possible association pattern linking behavioral participation, psychological resources, and emotional adjustment. Consistent with self-determination theory, PE may be associated with more adaptive coping beliefs and emotion-regulation capacities by providing mastery experiences, competence feedback, and peer interaction during exercise participation (Teixeira et al., 2012). Although PE may also be associated with PD through unmeasured pathways, such as physiological recovery, sleep improvement, or social connectedness, the nonsignificant PE → PD path in the moderated model suggests that its independent direct effect should be interpreted cautiously (Hird et al., 2024; Du et al., 2026). Overall, GSE, EI, and EI-related conditional indirect pathways more consistently accounted for the association between PE and PD. Accordingly, schools and families may consider regular physical exercise as a practical entry point for promoting mental health among rural left-behind adolescents, particularly those with characteristics similar to the present sample. By encouraging greater engagement in physical exercise, such efforts may help foster psychological resources such as GSE and EI, thereby being associated with lower PD and more balanced physical and psychological adjustment.

### 4.2 The mediating role of general self-efficacy

This study found that GSE significantly mediated the association between PE and PD among left-behind adolescent students. Among all indirect pathways, the pathway through GSE accounted for the largest proportion of the total effect (52.94%), which was substantially greater than the separate indirect effect through EI (9.29%). This finding suggests that GSE may serve as one of the more proximal psychological resources linking PE to PD. The protective association between PE and mental health among left-behind adolescent students may therefore extend beyond a direct reduction in negative emotions. Rather, PE may be linked to lower PD partly through internal psychological resources. This finding is in line with self-efficacy theory, which suggests that beliefs about one’s ability to manage challenges can shape emotional responses, stress tolerance, and psychological adjustment (Bandura, 1977). PE may be associated with higher GSE because exercise provides repeated chances for mastery, feedback, and self-confirmation. Through learning skills, reaching goals, and improving physical fitness, adolescents may report stronger competence beliefs about their own competence. These competence beliefs are conceptually related to GSE (Fossli and Lorentzen, 2025; Bandura, 1978). This association may be particularly relevant for left-behind adolescent students, who are more likely to experience weakened perceived control and lower coping confidence in the context of prolonged parental absence and limited emotional support. In such circumstances, GSE may serve as a cognitive resource, being associated with lower PD in the context of adverse experiences (Zhuang et al., 2025). Based on these findings, future school-based PE programmes may not need to focus solely on increasing activity participation, but could also consider supporting GSE. Structured goal setting, skill mastery, and positive feedback may be associated with stronger perceived competence and coping confidence, thereby providing possible psychological resource support associated with lower PD.

### 4.3 The mediating role of emotional intelligence

This study found that EI significantly mediated the association between PE and PD among rural left-behind adolescent students in China. However, the indirect effect through EI was relatively small, accounting for 9.29% of the total effect. This indicates that EI contributed to the PE–PD association, but its explanatory role was limited compared with other pathways. This pattern suggests that the PE–PD association may first be linked to GSE, while EI may provide an additional pathway related to emotion regulation. This finding is in line with prior evidence that EI is associated with lower negative emotional reactions and reduced psychological maladjustment. In the present study, EI may mainly help adolescents manage stress after it has occurred. Its value may not lie in removing stressors, but in improving adolescents’ ability to understand and regulate negative emotional experiences (Zhang et al., 2016). EI was examined as a mediator between PE and PD because PE is not limited to physical movement. It also involves social interaction and emotional experience. In PE settings, adolescents often need to follow rules, control their bodies, cooperate with peers, compete, and respond to success or failure. These situations may encourage them to recognize, express, and regulate emotions, which may be associated with higher EI (Li et al., 2024). This pathway may be particularly relevant for left-behind adolescent students. Parent–child separation may not directly lead to a lack of emotional ability, but it can create continuing emotional strain over time. In this context, EI may help students cope with negative experiences that have already occurred, rather than prevent stress at its source. Therefore, PE programs for left-behind adolescent students should not be limited to improving physical fitness. Schools and families could also include guidance on emotional awareness, expression, and regulation. By using PE situations that involve cooperation, competition, and frustration, these programs may help students develop EI and better cope with PD.

### 4.4 The serial mediating roles of general self-efficacy and emotional intelligence

One of the key findings of this study is that GSE and EI showed a significant serial mediating role in the association between PE and PD among rural left-behind adolescent students. The theoretical significance of this result lies not merely in placing previously established bivariate associations among PE, GSE, EI, and PD into a single model, but in suggesting that the association between PE and PD may involve a possible cognitive-emotional pathway through which PE is statistically linked to PD. The goal attainment, skill mastery, and peer interaction embedded in PE may not only provide immediate emotional release, but may also help individuals develop more positive coping expectations through repeated mastery experiences and competence feedback (Hagger, 2019). These coping expectations are linked to the cognitive competence belief represented by GSE, and higher GSE may make individuals more willing to notice, interpret, and regulate emotional experiences when facing stress and negative emotions. In this sense, EI in this pathway is not simply another general protective factor parallel to GSE, but may represent the further expression of cognitive competence beliefs at the level of emotional processing. Previous research has also shown a close association between GSE and EI in adolescent psychological adjustment (Salavera et al., 2017), which is consistent with the present findings.

This finding further suggests that the mental health relevance of PE may not operate through a single mediating resource alone. Previous studies have separately examined the associations between PE and GSE, between PE and EI, or between GSE/EI and stress adaptation. By placing these associations within a single model, the present study suggests that cognitive and emotional resources may not function as isolated mechanisms. Studies by Navarro-Mateu et al. (2020) and Udayar et al. (2020) also indicate close links among GSE, EI, and stress adaptation. Thus, the integrated model in this study offers a more specific theoretical perspective for understanding the association between PE and PD among rural left-behind adolescent students: the protective relevance of PE may be reflected in the linked cognitive and emotional psychological resources, rather than in the independent role of any single resource. Of course, this interpretation does not deny the possibility of alternative theoretical pathways. Individuals with higher EI may also develop stronger GSE through more effective emotional understanding and regulation, and GSE and EI may mutually reinforce each other during development. The present study selected the GSE → EI ordering primarily because it focuses on how mastery experiences and competence feedback provided by PE may first become linked to individuals’ cognitive competence beliefs and then be further expressed through the activation of emotional processing and regulation abilities.

In the context of left-behind adolescent students, this serial pathway also has practical explanatory value. Prolonged parent–child separation and limited emotional support may make these students more dependent on internal cognitive resources and proximal emotion-regulation resources to maintain adjustment (Zhuang and Wu, 2024). In this context, if individuals lack the basic competence belief that “I can cope,” even some emotional knowledge or regulation strategies may not be consistently applied in stressful situations. By contrast, higher GSE may be associated with stronger initiative and perceived control when facing stress, making individuals more willing to mobilize the emotional identification, understanding, and regulation abilities involved in EI. Broaden-and-build theory also supports this interpretation, suggesting that positive cognitive-emotional resources may be mutually related through repeated experience and may be associated with more stable adaptive resources (Fredrickson, 2001; Stanley and Schutte, 2023). Therefore, this study suggests that future school-based PE programmes could attend to the possible linkage between GSE and EI, rather than treating them as isolated targets. By supporting students’ competence expectations and emotional processing and regulation, the psychological relevance of PE may be more consistently linked to psychological resources associated with lower PD.

### 4.5 The moderating role of gender in the relationship between emotional intelligence and psychological distress

The results showed that, after gender and the EI × Gender interaction term were included, the direct effect of PE on PD was no longer significant. Statistically, this change can be interpreted as follows: once the model further accounted for the gender-specific emotional processing pathway, the remaining direct association between PE and PD may have been better accounted for by the cognitive-emotional resource pathway represented by GSE, EI, and EI × Gender. In other words, the association between PE and PD may not mainly reflect a robust residual direct association, but may instead operate through a psychological resource pathway characterized by “exercise participation–competence beliefs–emotional processing–psychological adjustment.”

This finding highlights the theoretical importance of the EI × Gender pathway. Previous research has shown gender differences in both EI and PD. Girls generally score higher than boys on overall EI and several of its branches (Megías-Robles et al., 2024). At the same time, they are more likely to report internalizing mental health problems, and this gender difference is particularly evident in PD (Campbell et al., 2021). These gender differences may be related to pubertal development, emotional sensitivity, and socialization processes. For example, pubertal development may shape adolescents’ emotional sensitivity and stress-response patterns (Kretzer et al., 2024; Milas et al., 2025), whereas gender-role expectations and socialization pressures may further reinforce gender differences in emotional expression and coping styles (Tamres et al., 2002; Ye and Pan, 2011). However, the focus of the present study is not whether gender affects the mean levels of EI or PD, but whether gender changes the strength of the association between EI and PD.

From the perspective of emotion-regulation strategies, girls are more likely to use emotion-focused coping and to engage in rumination under stress. A meta-analysis by Tamres et al. (2002) showed that women more frequently use coping strategies involving emotional expression and processing, including seeking emotional support, ruminating about problems, and positive self-talk. Nolen-Hoeksema (2012) also noted that women’s greater tendency toward rumination partly explains their higher risk of depression and anxiety. Thus, for girls, EI may not merely be a general positive resource, but a key protective factor against rumination, emotional dysregulation, and PD. Higher EI may help individuals identify, understand, and regulate negative emotions more accurately, thereby weakening the link between emotional processing burden and PD.

More importantly, these gender differences should be understood within the local cultural context of rural Chinese left-behind families. In rural family socialization, girls are often expected to be “sensible,” compliant, and attentive to others’ emotions, whereas boys are more commonly expected to be tough, independent, and less emotionally expressive. Long-term parental migration may further amplify these role differences: left-behind girls may assume greater responsibilities for emotional reassurance, family assistance, and self-restraint, while having limited channels for emotional expression. Research on rural left-behind children has also shown that parental migration reshapes children’s family roles, caregiving responsibilities, and everyday experiences, making gender differences more pronounced in left-behind contexts (Ye and Pan, 2011). For left-behind adolescent students who experience prolonged parent–child separation, limited emotional support, and greater exposure to negative peer interactions, proximal emotion-regulation resources may be especially important (Lin et al., 2025). Therefore, EI may show stronger protective relevance for girls because it is directly related to their ability to identify, understand, and regulate accumulated negative emotions. In practice, school-based efforts to promote mental health through PE may not need to focus only on exercise participation itself, but may also incorporate competence-building and training in emotional identification, understanding, and regulation, with particular attention to girls’ emotional processing needs in stressful contexts.

## 5 Limitations and future directions

The present study has several limitations. First, convenience sampling was used, and the sample was drawn only from rural left-behind adolescent students in Yunnan, Guizhou, and Guangxi, all of which are located in western China. Therefore, the findings should be interpreted primarily in relation to rural left-behind adolescent students in western China. They should not be directly generalized to rural areas in eastern, northern, or northwestern China, nor to urban left-behind children, non-left-behind rural adolescents, or students at other developmental stages. Future studies could use stratified random sampling or multi-region designs that include rural and urban adolescents from different economic, cultural, and educational contexts to further examine the external validity of the model.

Second, this study did not fully account for heterogeneity within left-behind experiences. Because parental migration pattern, duration of left-behind status, primary caregiver type, and frequency of parent–child contact were not measured, the current model cannot rule out the possibility that these left-behind characteristics confounded the associations among PE, GSE, EI, and PD. For example, adolescents with both parents away may differ from those with only one parent away, and short-term and long-term left-behind experiences may have different implications for psychological resources and psychological distress. Future research should systematically collect and control for these key variables to further examine whether the indirect pathways between PE and psychological distress remain stable.

Third, the cross-sectional design limits inference about temporal ordering and causality. The present study can only identify statistical associations among PE, GSE, EI, and PD, and cannot establish their temporal sequence or causal direction. In addition, the proposed GSE → EI serial ordering was mainly based on theoretical reasoning and prior literature, rather than on temporal precedence that could be directly demonstrated by cross-sectional data. Although this study conceptualized GSE as a more upstream cognitive competence belief and EI as a more downstream emotional processing and regulation ability, alternative theoretical models may also be plausible. The inability to compare these competing models is an important limitation. Future studies should use longitudinal, cross-lagged, random-intercept cross-lagged, or intervention designs to further examine the temporal ordering and possible reciprocal associations between GSE and EI.

Finally, all core variables were reported by the same participants at the same time point using self-report questionnaires. Although Harman’s single-factor test did not indicate severe common method bias, this test is relatively coarse and cannot fully rule out common method bias. In addition, the PARS-3 uses a multiplicative formula to calculate the PE total score, which may introduce non-normality and affect the stability of regression coefficients involving PE. Although the PE total score showed only mild positive skewness in the present sample, all continuous variables entered into the regression models were standardized, and indirect effects were estimated using bootstrap methods, the assumption of full multiplicativity among intensity, duration, and frequency embedded in the PARS-3 scoring method should still be interpreted cautiously. Future research could combine objective physical activity monitoring, teacher ratings, caregiver reports, or multi-wave measurement, and use more rigorous tests of common method bias. Future studies could also further compare the consistency of findings based on the original PARS-3 score, transformed scores, and objective physical activity indicators to strengthen the robustness of the conclusions.

## 6 Conclusion

In the present sample of rural left-behind adolescents, PE was significantly and negatively associated with PD. GSE and EI not only served as separate mediators in this association, but also formed a significant serial mediation pathway. Gender further moderated this association pattern, with the protective association between EI and PD, as well as EI-related indirect effects, being stronger among female students. Given the cross-sectional design, these findings should be interpreted as associative rather than causal. They provide preliminary empirical clues and a theoretical framework for future PE-based mental health promotion programmes for left-behind adolescents, particularly programmes that consider cognitive competence beliefs, emotional processing abilities, and gender differences. Longitudinal and experimental studies are still needed to verify whether PE contributes to subsequent changes in GSE, EI, and PD and to examine whether these associative pathways can inform future programme design.

## Data Availability

The datasets generated and/or analyzed during the current study are available from the corresponding author upon reasonable request.
